# Pressured-induced superconducting phase with large upper critical field and concomitant enhancement of antiferromagnetic transition in EuTe_2_

**DOI:** 10.1038/s41467-022-30718-5

**Published:** 2022-05-27

**Authors:** P. T. Yang, Z. Y. Liu, K. Y. Chen, X. L. Liu, X. Zhang, Z. H. Yu, H. Zhang, J. P. Sun, Y. Uwatoko, X. L. Dong, K. Jiang, J. P. Hu, Y. F. Guo, B. S. Wang, J.-G. Cheng

**Affiliations:** 1 Beijing National Laboratory for Condensed Matter Physics and Institute of Physics, Chinese Academy of Sciences, Beijing, 100190 China; 2 School of Physical Sciences, University of Chinese Academy of Sciences, Beijing, 100190 China; 3 School of Physical Science and Technology, Shanghai Tech University, Shanghai, 201210 China; 4 Songshan Lake Materials Laboratory, Dongguan, Guangdong, 523808 China; 5 Institute for Solid State Physics, University of Tokyo, Kashiwa, Chiba, 277-8581 Japan

**Keywords:** Superconducting properties and materials, Magnetic properties and materials

## Abstract

We report an unusual pressure-induced superconducting state that coexists with an antiferromagnetic ordering of Eu^2+^ moments and shows a large upper critical field comparable to the Pauli paramagnetic limit in EuTe_2_. In concomitant with the emergence of superconductivity with *T*_c_ ≈ 3–5 K above *P*_c_ ≈ 6 GPa, the antiferromagnetic transition temperature *T*_N_(*P*) experiences a quicker rise with the slope increased dramatically from d*T*_N_/d*P* = 0.85(14) K/GPa for *P* ≤ *P*_c_ to 3.7(2) K/GPa for *P* ≥ *P*_c_. Moreover, the superconducting state can survive in the spin-flop state with a net ferromagnetic component of the Eu^2+^ sublattice under moderate magnetic fields *μ*_0_*H* ≥ 2 T. Our findings establish the pressurized EuTe_2_ as a rare magnetic superconductor possessing an intimated interplay between magnetism and superconductivity.

## Abstract (web summary)

Here, the authors report pressure-induced superconductivity with concomitant enhancement of antiferromagnetic transition in layered EuTe_2_. The superconductivity is distinctly characterized by the high upper critical fields exceeding the Pauli limit among binary tellurides, a prerequisite of the coexistence of ferromagnetism with superconductivity.

## Introduction

The interplay between static magnetism and conduction electrons has been a core topic in modern condensed matter physics spanning a broad spectrum of interesting phenomena including the Kondo physics^1^ and heavy fermions^2^, dilute magnetic semiconductors^3^, giant/colossal magnetoresistance (MR)^4^, and spintronics^5^. Recently, the addition of non-trivial band topology to this topic leads to more exotic topological quantum phenomena such as quantum anomalous Hall effect and the intrinsic large anomalous Hall effect^6–8^. When the conduction electrons condense into Cooper pairs and coexist with static magnetism in the magnetic superconductors, unconventional pairing states with intriguing superconducting properties can emerge as exemplified by the U- and Eu-based magnetic superconductors^9–15^. Due to the antagonistic nature between magnetism and superconductivity, however, the magnetic superconductors are rare^16–18^, and the concurrence of above-mentioned phenomena in a single material is even scarce. Here we report on a rare case that manifests an intimated interplay among static magnetism, conduction electrons, and possible exotic superconductivity through pressure regulations on an antiferromagnetic (AF) semiconductor EuTe_2_.

**Fig. 1:**
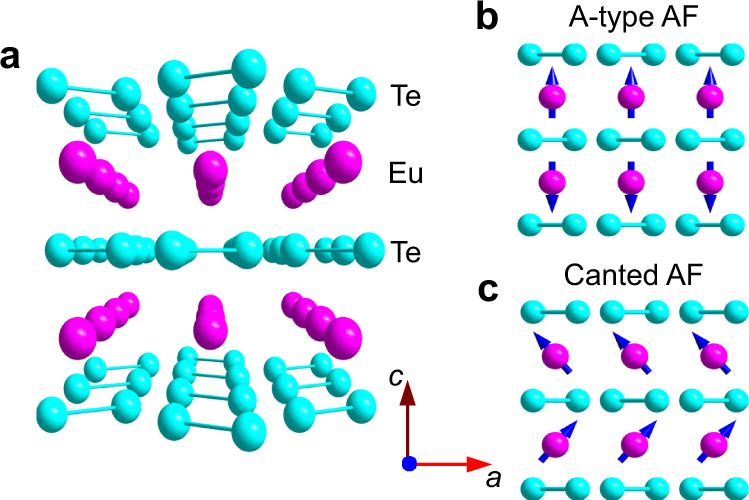
Crystal and magnetic structures of EuTe_2_. **a** Schematic view of the layered crystal structure of EuTe_2_, **b** Type-A antiferromagnetic (AF), and **c** Canted AF structure of Eu^2+^ spins in EuTe_2_.

At ambient pressure (AP), EuTe_2_ crystallizes in a CuAl_2_-type tetragonal structure with space group I4/mcm (No. 140), Fig. 1a. Each Eu^2+^ ion is surrounded by eight Te atoms, which form the [Te_2_]^2−^ dimers stacking along the *c*-axis^19^. Upon cooling down at zero field, EuTe_2_ exhibits a semiconducting behavior in resistivity *ρ*(*T*) and the Eu^2+^ moments develop a type-A AF order below *T*_N_ = 11 K, having the *c*-axis-oriented ferromagnetic (FM) Eu^2+^ layers coupled antiferromagnetically as depicted in Fig. 1b. When an external magnetic field is applied along *c*-axis, the type-A AF order can be tuned into a canted AF state, Fig. 1c, through a spin-flop transition at *μ*_0_*H*_1_ = 2.3 T and then a fully spin-polarized state at *μ*_0_*H*_2_ = 7.6 T. Interestingly, these field-induced transitions have a profound impact on the transport properties of EuTe_2_; i.e., *ρ*(*T*) under *μ*_0_*H* > *μ*_0_*H*_1_ is altered from semiconducting to metallic-like behavior below a characteristic temperature *T*_m_ ≫ *T*_N_, resulting in a large negative MR with over five orders of drop in resistivity at low temperatures, Supplementary Fig. 1. According to the density-functional-theory calculations, the charge carriers near the Fermi level originate mainly from the Te-5p orbitals and the small energy gap of *E*_a_ ≈ 16 meV at zero field can be closed by lifting the band degeneracy of the Te-5p orbitals in the spin-flop state with a net FM component. This explains the field-induced metallic state and thus the large negative MR below *T*_m_^19^. Because the band structure of EuTe_2_ was found to depend strongly on the spin directions, it has been proposed as a promising material platform to develop novel electronic devices^20,21^. These results thus demonstrated an intimated interplay between the static magnetism of Eu^2+^ sublattice and the charge carries from Te-5p orbitals in EuTe_2_.

Since EuTe_2_ is characterized as a small-gap AF semiconductor, its electrical transport and magnetic properties are expected to be tuned effectively by applying high pressures. Considering the fact that many Te-containing materials become superconducting at ambient and/or high pressures (Table 1), it is imperative to pursue whether EuTe_2_ can be driven into a magnetic superconductor upon compression. To this end, we are motivated to investigate the effect of pressure on the transport and magnetic properties of EuTe_2_ single crystals by using a cubic anvil cell (CAC) apparatus^22,23^.

**Table 1:** Summary of the superconducting transition temperature *T*_c_ and the upper critical fields *μ*_0_*H*_c2_(0) for the reported Te-containing superconductors.

Compound	*T*_c_ (K)	*μ*_0_*H*_c2_(0) (T)	*μ*_0_*H*_p_(0) (T)	*H*_c2_(0)*/H*_p_(0)	*P* (GPa)
EuTe_2_^This work^	3.71	8.02	6.83	1.175	6.6
Te^44^	2.2	0.032	4.05	0.008	3.7
	4.3	0.065	7.91	0.008	5.2
GdTe_3_^36^	0.55	0.018	1.01	0.018	1.2
	0.87	0.076	1.60	0.047	1.8
	1.13	0.14	2.08	0.067	2.5
TbTe_3_^36^	1.75	0.026	3.22	0.008	0.84
DyTe_3_^36^	0.73	0.018	1.34	0.013	1.2
	0.92	0.035	1.69	0.021	1.8
	1.2	0.061	2.21	0.028	2.5
ErTe_3_^37^	2.7	~0.05	4.97	~0.010	AP
Au_0.65_Pt_0.35_Te_2_^45^	4.0	~1.29	7.36	~0.175	AP
ZrTe_3_^46^	4.8	5.13	8.83	0.581	AP
HfTe_3_^47^	1.65	~0.6	3.04	~0.197	AP
HfTe_5_^48^	4.8	4.5	8.83	0.510	19.5
MoTe_2_^49^	8.2	4.0	15.09	0.265	11.7
WTe_2_^50^	6.0	2.72	11.04	0.246	24.6
CuTe^51^	3.0	0.26	5.52	0.047	43.7
CeTe_1.82_^52^	2.7	~0.5	4.97	~0.101	0.5
NiTe_2-x_^53^	4.4	0.125	8.10	0.015	3.8
	7.8	0.08	14.35	0.006	51
Bi_2_Te_3_^54^	3.0	1.8	5.52	0.326	6.1
Sb_2_Te_3_^55^	3.0	2.6	5.52	0.471	6.7
Bi_1.1_Sb_0.9_Te_2_S^56^	4.0	2.31	7.36	0.314	14.3
Sn_0.8_In_0.2_Te^57^	3.3	1.33	6.07	0.219	AP
Ir_0.93_Te_2_^38^	4.7	4.58	8.65	0.530	AP
TiTe_2_^39^	5.7	5.5	10.49	0.524	24.3
CrSiTe_3_^40^	4.0	4.0	7.36	0.543	19.5
Ba_3_TiTe_5_^58^	4.0	8.0	7.36	1.087	17.3
CsBi_4_Te_6_^59^	4.4	9.7	8.10	1.198	AP
UTe_2_^16^	1.6	>40	2.94	>13.587	AP
FeTe_0.8_S_0.2_^60^	10.0	~70	18.40	~3.804	AP

We find that the application of high pressure drives EuTe_2_ from an AF semiconductor showing a large negative MR into a magnetic superconductor with a large upper critical field comparable to the Pauli paramagnetic limit. Moreover, the emergence of superconductivity above the critical pressure of *P*_c_ ≈ 6 GPa is accompanied with a concomitant enhancement of *T*_N_, and the superconductivity can survive in the spin-flop state with a net FM component under moderate fields *μ*_0_*H* ≥ 2 T, implying a possible exotic pairing state. These new results under high pressures, especially the discovery of superconductivity coexisting with magnetic order, have enriched the physics pertinent to the interplay between static magnetism and conduction electrons in this interesting compound.

## Results

### High-pressure resistivity

**Fig. 2:**
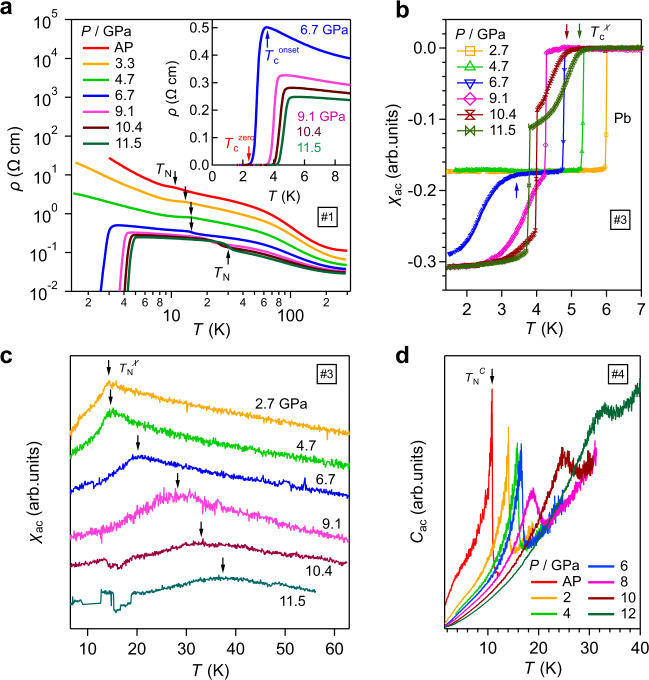
Physical properties of EuTe_2_ under high pressures. Temperature dependence of **a** resistivity *ρ*(*T*) for sample #1, **b**, **c** ac magnetic susceptibility *χ*_ac_(*T*) for sample #3, and **d** ac specific heat *C*_*ac*_(*T*) for sample #4 under various pressures up to 12 GPa. Inset of **a** shows the enlarged view of low-*T ρ*(*T*) data. The antiferromagnetic transition temperatures *T*_N_ and the superconducting transition temperatures *T*_c_^zero^ and *T*_c_^onset^ are marked by arrows in these plots.

Figure 2a shows the *ρ*(*T*) in a double-logarithmic plot for sample #1 under various pressures up to 11.5 GPa. The semi-logarithmic plot of *ρ*(*T*) and the temperature derivative, d*ρ*/d*T*, are displayed in Supplementary Fig. 2. Similar to the previous report^19^, the *ρ*(*T*) at AP exhibits a semiconducting behavior in the whole temperature range, and the AF transition is manifested as a weak kink-like anomaly in *ρ*(*T*) at *T*_N_ ≈ 11 K, which can be determined from the minimum of d*ρ*/d*T*, Supplementary Fig. 2b. With increasing pressure, *ρ*(*T*) decreases monotonously in the whole temperature range but the main features are retained up to 4.7 GPa, and the kink-like anomaly at *T*_N_ moves to higher temperatures gradually. These observations indicate that pressure enhances the electrical conductivity and strengthens the AF interactions in EuTe_2_ as expected. At 6.7 GPa, the semiconducting *ρ*(*T*) remains at high temperatures, but the feature at *T*_N_ has been changed to a step-like anomaly, implying the modifications of either AF order or its interplay with conduction electrons. At this pressure, we surprisingly observed a sudden drop of resistivity that starts at *T*_c_^onset^ ≈ 3.4 K and reaches zero at *T*_c_^zero^ ≈ 2.4 K, inset of Fig. 2a, signaling the occurrence of superconductivity. When applying pressures gradually up to 11.5 GPa, both the step-like anomaly at *T*_N_ and the superconducting transition are shifted to higher temperatures progressively. At 11.5 GPa, these transition temperatures have been raised to *T*_N_ ≈ 30 K, *T*_c_^onset^ ≈ 5 K, and *T*_c_^zero^ ≈ 4.1 K, respectively. The observations of pressured-induced superconductivity and concomitant enhancement of *T*_N_ are reproduced in a separate run of resistivity measurements on sample #2, Supplementary Fig. 3. For both samples, although the normal-state *ρ*(*T*) is reduced considerably by pressure, it retains a semiconducting behavior in the investigated pressure/temperature ranges. This feature leads to an interesting finding that the superconductivity in EuTe_2_ emerges from a non-metallic normal state that should possess a rather low carrier density.

### AC magnetic susceptibility and ac specific heat

Because it is difficult to measure the Meissner effect directly under pressures much higher than 1 GPa, it is a common practice to estimate the superconducting volume fraction by measuring ac magnetic susceptibility *χ*_ac_(*T*) with the mutual induction method under high pressures. In this way, filamentary superconductivity arising from minor impurity or superconductivity with a rather low volume fraction can be easily excluded. In addition, pressure-induced gradual growth of superconducting phase at the expense of other competing phases can also be revealed as shown in our previous work^24^. Thus, this method is proved to be reliable in confirming bulk superconductivity as long as the sample is not covered uniformly by a superconducting layer. To exclude such a possibility, we polished carefully the sample surfaces before high-pressure measurements in the present study.

Figure 2b displays the *χ*_ac_(*T*) data measured on sample #3 together with a piece of lead (Pb) under various pressure up to ~12 GPa in a CAC. The superconducting shielding volume fraction can be estimated by comparing the diamagnetic signals of EuTe_2_ and Pb. As shown in Fig. 2b, the *χ*_ac_(*T*) at 2.7 and 4.7 GPa exhibit only one sharp drop corresponding to the superconducting transition of Pb, which moves to lower temperatures gradually with increasing pressure. The *χ*_ac_(*T*) at 6.7 GPa displays an additional, relatively broad diamagnetic signal at *T*_c_^χ^ ≈ 3.5 K, which agrees well with the *T*_c_^onset^ determined from *ρ*(*T*). In line with the above *ρ*(*T*) results, *T*_c_^χ^ also moves to higher temperatures gradually with pressure and the superconducting shielding volume fraction reaches about unity by comparing the diamagnetic signals of EuTe_2_ with that of Pb. Such a perfect diamagnetic response and the large upper critical field shown below exclude the possibility that the observed superconductivity comes from the Te impurity and confirm the bulk nature of the observed superconductivity.

The variation with the pressure of *T*_N_ is also confirmed by the *χ*_ac_(*T*) and ac specific heat *C*_ac_(*T*) measurements. As shown in Fig. 2c, d, the AF transition is manifested as a cusp-like anomaly in *χ*_ac_(*T*) and a sharp λ-shaped peak in *C*_*ac*_(*T*) at low pressures. The obtained transition temperatures match well to those determined from *ρ*(*T*) and shifts to higher temperatures progressively with increasing pressure. It is noteworthy that the corresponding anomalies in *χ*_ac_(*T*) and *C*_*ac*_(*T*) around *T*_N_ are significantly broadened up for *P* > 6 GPa, which might be attributed to the reduction of Eu^2+^ moments due to pressure-induced valence change^25,26^. Based on the above results, we can reach the conclusion that bulk superconductivity emerges above *P*_c_ ≈ 6 GPa within the antiferromagnetically ordered state of EuTe_2_, thus making it a magnetic superconductor.

It is noted that we failed to observe any anomaly in the *C*_*ac*_(*T*) around *T*_c_. Some plausible reasons are as follow: (1) the superconductivity in EuTe_2_ develops from a non-metallic normal state which should be featured by a rather low carrier density, and thus the superfluid density is low; (2) the superconductivity emerges deep inside an A-type AF phase of large-moment Eu^2+^ spins, i.e*. T*_c_ ≪ *T*_N_, then the exchange field produces a vortex state in the absence of external magnetic field so that the jump in *C*_*ac*_(*T*) around *T*_c_ has been considerably smeared out. For the same reason, the specific-heat jump around *T*_c_ can be hardly discerned in the magnetic superconductor Eu(Fe_1-x_Ni_x_)_2_As_2_ even at AP^27^.

### Magneto-transport properties under pressure

**Fig. 3:**
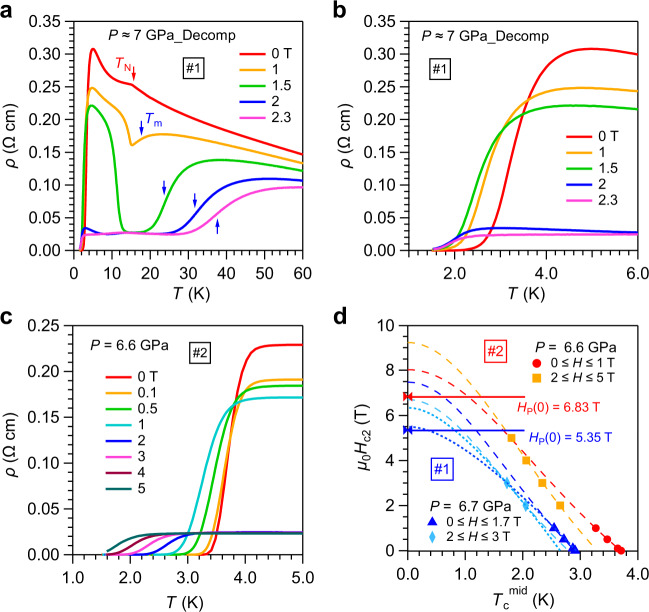
Magneto-transport properties of EuTe_2_. *ρ*(*T*) of sample #1 at ~7 GPa (Decompression) under various fields in the temperature range of **a** 0–60 K and **b** 1–6 K. **c** Low-temperature *ρ*(*T*) of sample #2 at 6.6 GPa under various fields. **d** Temperature dependence of the upper critical field *μ*_0_*H*_c2_ for sample #1 at 6.7 GPa and sample #2 at 6.6 GPa, respectively. The broken and dotted lines in **d** represent the fitting curves by using the Ginzburg–Landau (G–L) and Werthamer–Helfand–Hohenberg (WHH) models, respectively. The Pauli-limiting fields *H*_p_(0) are given and indicated by the horizontal lines in **d**.

**Fig. 4:**
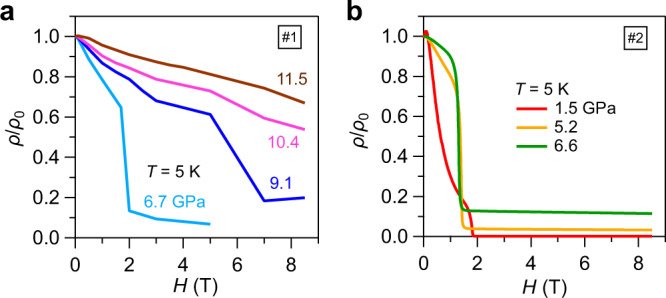
Effect of pressure on the spin-flop transition. Field dependence of normalized resistivity *ρ/ρ*_0_(*H*) at 5 K under different pressures for **a** sample #1 and **b** sample #2. The sudden drop of *ρ/ρ*_0_ is associated with field-induced spin-flop transition.

To characterize the AF and superconducting states under pressures, we measured the magneto-transport properties of EuTe_2_ at different pressures. Figure 3a presents the *ρ*(*T*) data below 60 K for sample #1 under various magnetic fields at *P* ≈ 7 GPa. These data were collected during the decompression process and the pressure value of ~7 GPa was estimated by comparing the room-temperature resistivity value to that during the compression process. Thus, we have labeled the pressure as “7 GPa_Decomp”. The kink-like anomaly at *T*_N_ and the superconducting transition is clearly visible in *ρ*(*T*) at 0 T. Upon cooling down under fields *μ*_0_*H* < 2 T, the semiconducting behavior at the normal state is replaced by a metallic-like behavior (d*ρ*/d*T* > 0) below *T*_m_ and then a re-entrant semiconducting phase (d*ρ*/d*T* < 0). With increasing field, the metallic region is expanded at the expense of the re-entrant semiconducting phase, and finally a metallic, low-resistance state below *T*_m_ is achieved at *μ*_0_*H* > 2 T due to the spin-flop transition, which causes a sudden drop in the field-dependent resistivity at 5 K as seen in Fig. 4. From Fig. 3a, we can see that *T*_m_ shifts to higher temperatures quickly with increasing magnetic fields. All these features in magneto-transport properties resemble those at AP, Supplementary Fig. 1, indicating that the type-A AF structure of EuTe_2_ should be retained under pressures at least up to 7 GPa.

To our surprise, the superconducting state dominated by Te-5p electrons can survive at *μ*_0_*H* ≥ 2 T in the spin-flop state, when the canted Eu^2+^ moments contribute to a large FM component, Fig. 1c. The low-temperature *ρ*(*T*) data at ~7 GPa for sample #1 and at 6.6 GPa for sample #2, Fig. 3b, c, illustrates the evolution of the superconducting transition under various magnetic fields. As expected, *T*_c_ is suppressed to the lower temperatures by magnetic field owing to the pair-breaking effect. The normal-state resistivity first decreases gradually and then drops abruptly at ~2 T associated with the spin-flop transition. We measured the field dependence of superconducting transition at each pressure and showed all the data in Supplementary Fig. 4. As can be seen, the drop of normal-state resistivity is reduced while the corresponding critical field is enhanced progressively with increasing pressure, which should be attributed to the modification of AF structure under pressures. As seen in Fig. 4a, the field-induced spin-flop transition becomes much weaker and is almost diminished at pressures above 10.4 GPa.

### The upper critical field

To quantify the evolution of the superconducting transition, especially around *P*_c_, we have determined *T*_c_^mid^ according to the criteria of 50% *ρ*_n_ from the *ρ*(*T*) data in Fig. 3b, c and Supplementary Fig. 4a–d, and plotted the obtained *μ*_0_*H*_c2_ versus *T*_c_^mid^ in Fig. 3d and Supplementary Fig. 4e. As seen in Fig. 3d, the experimental points for both samples do not fall onto a single line, but experiences a side-jump shift around 2 T. This feature originates from field-induced spin-flop transition that contributes to an additional internal field (*μ*_0_*H*_int_ on the order of ~1 T depending on the canting angle) and thus lowers *T*_c_. In the presence of additional *μ*_0_*H*_int_ at *μ*_0_*H* ≥ 2 T, surprisingly, the slope of *μ*_0_*H*_c2_(*T*) becomes even larger for sample #2 as seen in Fig. 3d. This means that the superconducting state can resist higher magnetic fields. But the opposite is observed for sample #1. We attributed such a difference to the different orientations of magnetic field with respect to the *c*-axis and will clarify it in the future study.

In our experimental setup employing an L-He^4^ cryostat and a 9 T superconducting magnet, the lowest temperature that we can reach is about 1.5 K in the presence of a large cubic-anvil pressure cell. Since the *T*_c_ of EuTe_2_ under pressures falls in the range of 3–5 K, we can merely access a limited temperature range of 0.5 ≤ *T*/*T*_c_ ≤ 1 in the *μ*_0_*H*_c2_-*T* phase diagram, which prevents us from obtaining a complete *H*_c2_(*T*) curve in the present study. As such, we had to resort to empirical Ginzburg–Landau (G–L) fitting to extrapolate the zero-temperature values of *μ*_0_*H*_c2_(0), viz., *μ*_0_*H*_c2_(*T*) = *μ*_0_*H*_c2_(0)[1−(*T*/*T*_c_)^2^]/[1 + (*T*/*T*_c_)^2^]^28^. The best fits shown as the broken lines in Fig. 3d yield the *μ*_0_*H*_c2_(0) values of 7.5 T (8.0 T) for *μ*_0_*H* < 2 T and 6.7 T (9.2 T) for *μ*_0_*H* > 2.0 T for sample #1 (#2), respectively. Accordingly, the coherence lengths of *ξ*(0) = 66.2 Å (64.1 Å) and 70.1 Å (59.8 Å) can be obtained for the low- and high-field regions, respectively, for sample #1 (#2) according to the equation *μ*_0_*H*_c2_(0) = *Φ*_0_/2*πξ*(0)^2^ where *Φ*_0_ = 2.067 × 10^−15 ^Wb is the magnetic flux quantum^29^. These differences demonstrate a modification of the superconducting vortex state by field-induced spin-flop transition of Eu^2+^ sublattice. Moreover, both *μ*_0_*H*_c2_(0) values are larger than the Pauli paramagnetic limit^30^, i.e. *μ*_0_*H*_p_ = 1.84*T*_c_ indicated by the solid lines in Fig. 3d. For *P* > *P*_c_, when the impact of spin-flop transition is weakened in the studied field range, the *μ*_0_*H*_c2_(*T*) can be described by a single G-L fitting curve, yielding *μ*_0_*H*_c2_(0) values still above the Pauli limit, as seen in Supplementary Fig. 4e.

Since the above G–L fitting in a limited temperature range can give a relatively large uncertainty of *μ*_0_*H*_c2_(0), we also estimated the orbital limiting *μ*_0_*H*_c2_^orb^(0) based on the Werthamer–Helfand–Hohenberg (WHH) model^31^. As shown by the dotted lines in Fig. 3d and Supplementary Fig. 5, the obtained *μ*_0_*H*_c2_^orb^(0) values are smaller than those estimated from the G–L fitting, but they are still large, decreasing gradually from larger than *μ*_0_*H*_p_ at 6.7 and 9.1 GPa to slightly smaller than *μ*_0_*H*_p_ at 10.4 and 11.5 GPa. These results indicate that the presence of a large *μ*_0_*H*_c2_(0) is an intrinsic characteristic of the superconducting state of EuTe_2_ under pressures. This is in strikingly contrast to those superconductors derived from Te-5p electrons that usually possess *μ*_0_*H*_c2_(0) about two orders of magnitude smaller as shown in Table 1. In addition to the possible unconventional pairing mechanism, other factors such as the strong coupling effect can also be invoked to explain the observed large *μ*_0_*H*_c2_(0).

### Temperature-pressure and temperature-field phase diagrams

**Fig. 5:**
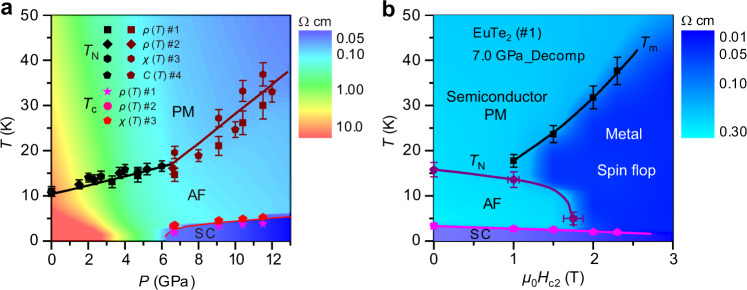
Phase diagrams of EuTe_2_. **a** Temperature-pressure phase diagram of EuTe_2_ based on the *ρ*(*T*), *χ*_ac_(*T*), and *C*_*ac*_(*T*) data. **b** Temperature-field phase diagram of EuTe_2_ (#1) at ~7 GPa based on the *ρ*(*T*) data in Fig. 3a. PM, AF, and SC denote paramagnetic, antiferromagnetic, and superconducting phases, respectively. The color indicates the magnitude of resistivity. The error bars of the transition temperatures in (**a**, **b**) are estimated from the width of the transitions.

The above results can be visualized vividly from the pressure- and field-dependent phase diagrams of EuTe_2_, as shown in Fig. 5a, b, which highlights the main findings of this work: (i) *T*_N_(*P*) increases with pressure and the slope d*T*_N_/d*P* is enhanced significantly from 0.85 K/GPa for *P* ≤ *P*_c_ to 3.7 K/GPa at *P* ≥ *P*_c_; (ii) bulk superconductivity emerges above *P*_c_ ≈ 6 GPa, in concomitant with the quick rise of *T*_N_(*P*), and *T*_c_(*P*) also increases with pressure; (iii) the normal-state *ρ*(*T*) above *T*_c_ retains a semiconducting behavior up to 12 GPa; (iv) the superconductivity survives in a wide field range inside both type-A AF and the spin-flop state with a net FM component because it possesses a large upper critical field comparable to the Pauli paramagnetic limit; (v) the superconducting state around *P*_c_ experiences a subtle modification accompanying the spin-flop transition. These results, especially the coexistence and mutual promotion of AF transition and superconductivity, are interesting and should be closely related with several key issues as discussed below.

## Discussion

### Structural stability under pressure

**Fig. 6:**
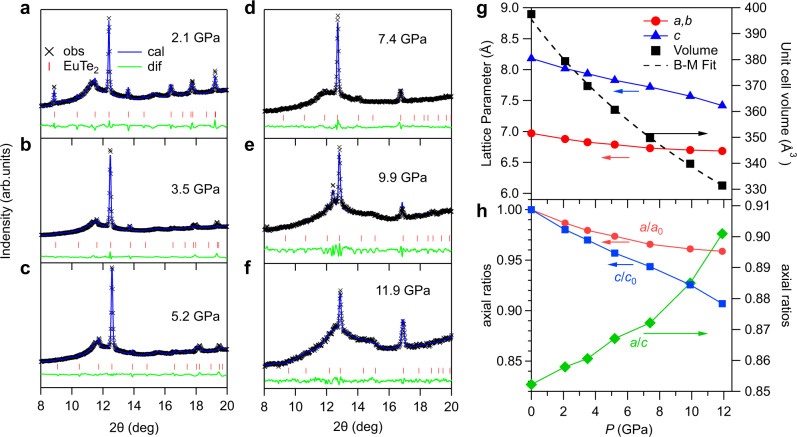
Structural evolutions of EuTe_2_ under high pressure. **a**–**f** High-pressure synchrotron X-ray diffraction under various pressures up to 11.9 GPa at room temperature. **g**, **h** Pressure dependence of lattice parameters (*a*, *b*, *c*, volume *V*) and the relative shrinkage of lattice parameters *a*/*a*_0_, *c*/*c*_0_ and the axis ratio *a*/*c*. In **g**, the Birch–Murnaghan (B–M) isothermal equation of state was adopted to fit the pressure dependence of volume *V*(*P*). A clear slope change in *a*/*a*_0_, *c*/*c*_0_, and *a*/*c* can be found around 7 GPa.

We performed high-pressure X-ray diffraction (XRD) on pulverized EuTe_2_ samples up to 11.9 GPa. Figure 6a–f shows the XRD patterns after LaBeil fitting in the tetragonal structure with space group I4/mcm. As can be seen, the intensity of the main peak around 12.5° is reduced gradually and the relative intensity of other peaks also experiences some modifications with increasing pressure, presumably due to the presence of shear stress and/or reorientation of grains upon compression. The absence of clear peak splitting and the smooth contraction of lattice parameters with pressure indicates the absence of significant structural phase transition in the studied pressure range. It should be noted that the relatively low quality of our XRD data prevented us from refining the atomic positions of Te atoms (the position of Eu at 4a site is fixed) under pressures. Thus, the possibility of a weak, hidden structural transition associated with the change of Te positions cannot be ruled out within the resolution of our XRD data. Further studies on high-quality XRD data are needed to verify this scenario.

The lattice compressibility of a compound is mainly determined by the chemical bonding characters of the crystal structure, and the structural responses to pressure will then influence the spin-spin exchange interactions. Vice versa, we think that the magnetic exchange interactions can also influence the lattice compressibility in the layered magnetic materials. As shown in Fig. 6g, h, the unit-cell parameters exhibit strong anisotropic compressibility, i.e., from AP to 11.9 GPa, the *a* and *c* axis shrink by −4.1% and −9.3%, respectively. In addition, the lattice constant *a* becomes less compressible above *P*_c_, whereas the *c*-axis shows the opposite trend, resulting in a quicker rise of the *a/c* ratio. For EuTe_2_, the strong anisotropic compressibility should be attributed mainly to the layered structure as commonly seen in the layered magnetic materials such as Cr_2_Ge_2_Te_6_^32,33^. To rationalize this latter observation, in addition to the possible hidden structural transition mentioned above, we argued that the spin–spin interactions could influence the lattice compressibility. As we know, the magnetic striction around *T*_N_ (*T*_c_) is usually negative (positive) for the AF (FM) insulators [see, for example, the AF MnO and MnS^34^ and FM CrTe^35^]. Thus, a positive (negative) pressure effect on *T*_N_ (*T*_c_) is usually observed because a smaller-volume phase is favored under pressure. Although the spins remain in the paramagnetic state at room temperature, we deduce that the intralayer FM spin-spin interactions would resist the further contraction of *a*-axis while the interlayer AF interactions favor the contraction of *c*-axis. This can reconcile the observed opposite trend of compressibility along the *a-* and *c*-axis above 6 GPa. Accordingly, the reduction of unit-cell volume can strengthen the AF interactions, giving rise to a positive response of *T*_N_(*P*). However, this effect alone cannot explain the quicker rise of *T*_N_(*P*) above 6 GPa. Instead, other factors such as the enhanced coupling between spin and charge carriers might play an important role.

### Origin of superconductivity and concurrent enhancement of *T*_N_

According to band structure calculations at AP, the Eu-4f electrons reside ~2 eV below the Fermi level, while the Te-5p orbitals are much spread out in energy and make a weak contribution to the density of states at Fermi level^19,20^. Thus, EuTe_2_ can be regarded as a simple system in which Eu^2+^ contributes localized 4 f magnetic moments forming the AF order, while the Te-5p electrons dominate the charge transport. It should be the effects of spin-orbit coupling and electron correlations that open a small gap of the Te-5p bands near Fermi level, giving rise to a semiconducting behavior at AP. It is expected that high pressure can broaden up the Te-5p bandwidth and thus enhance its contributions near Fermi level. Indeed, the magnitude of resistivity, especially at low temperatures, is reduced progressively by pressure. Although the semiconducting behavior in the normal state is robust against pressure, the activation gap *E*_a_ extracted from fitting to the thermal activation model *ρ*(*T*) = *ρ*_0_exp(*E*_a_/*k*_B_*T*) decreases quickly from ~23 meV at AP to ~12.2 meV at *P*_c_, and then slowly to ~7 meV at 11.5 GPa, as shown in Supplementary Fig. 6. In this regard, it is reasonable to attribute the observed superconductivity in EuTe_2_ to the Te-5p electrons near Fermi level which become sufficient to form Cooper pairs at *T* < *T*_c_ and *P* > *P*_c_. Meanwhile, the *T*_N_(*P*) of Eu^2+^ sublattice exhibits a coincident quicker rise upon further increasing pressure. We believe that these two phenomena should be related to pressure-induced enhancement of charge carriers derived from the Te-5p bands. These charge carriers not only provide additional pathways mediating the magnetic exchange interactions between the Eu^2+^ spins, leading to a quicker rise of *T*_N_(*P*), but also form Cooper pairs at *T*_c _≪ *T*_N_. In this sense, the emergence of superconductivity is intimately correlated with the concomitant enhancement of *T*_*N*_(*P*) above *P*_c_. The observation of an unusually large *μ*_0_*H*_c2_(0) in the superconducting state that can survive in the spin-flop state with a net FM component indicates a possible unconventional pairing mechanism. Further first-principles calculations under pressure are needed to gain a better understanding of the peculiar properties of EuTe_2_.

### Unusually large upper critical field

The observed large *μ*_0_*H*_c2_(0) comparable to *μ*_0_*H*_p_ for the superconducting state in EuTe_2_ is unexpected. We have surveyed in Table 1 the *μ*_0_*H*_c2_(0) values for many Te-containing superconductors for comparison. For pure Te and other Te-dominated superconductors, tiny values of *μ*_0_*H*_c2_(0) are universally observed, e.g. *μ*_0_*H*_c2_(0) ≈ 0.065 T for the element Te at 5 GPa with a similar *T*_c_ = 4.3 K as seen in Supplementary Fig. 7, and *μ*_0_*H*_c2_(0) ≈ 0.02–0.07 T for some rare-earth tellurides *R*Te_3_ (*R* = Gd, Tb, Dy, Er) under pressures^36,37^. These values are about two orders lower than that of EuTe_2_. Although other Te-containing superconductors possess an *μ*_0_*H*_c2_(0) on the order of several Tesla, their density of states near Fermi level forming Cooper pairs have major contributions from d-orbitals for the transition-metal tellurides^38–40^ or f-orbitals for UTe_2_^16^ rather than the Te-5p electrons. It thus becomes an important issue in future studies to uncover the origin of such a large *μ*_0_*H*_c2_(0) in EuTe_2_.

For type-II superconductors, orbital and spin-paramagnetic effects are two main mechanisms of pair-breaking under magnetic fields. The former is related to the formation of Abrikosov vortex and the orbital-limiting field *μ*_0_*H*_c2_^orb^(0) refers to the critical field at which vortex cores begin to overlap, while the latter comes from the Zeeman splitting of spin-singlet Cooper pairs with the Pauli-limiting field *μ*_0_*H*_p_(0) = *g*^−1/2^*Δ*/*μ*_B_ in the Chandrasekhar-Clogston limit. For a weakly coupled BCS superconductor where 2*Δ*(0) = 3.52*k*_B_*T*_c_, the Pauli-limiting field is approximately *H*_p_^BCS^(0) = 1.84*T*_c_. The Maki parameter *α* = $\sqrt{2}$*H*_c2_^orb^(0)/*H*_p_(0) measures the relative importance between the orbital and spin-paramagnetic pair-breaking effect. The larger *α*, the more influence of Pauli-limiting effect. Since *α* is known to be the order of *Δ*(0)/*E*_F_, *α* is usually ≪1. In the present case, although the electron correlations are expected to be relatively weak, it may contain very small Fermi pockets given the non-metallic normal state above *T*_c_. As such, *E*_F_ can become quite small and thus results in *α* ≥ 1. To prove this point, we need to extend the *μ*_0_*H*_c2_(*T*) to much lower temperatures. Usually, the flattened behaviors of *μ*_0_*H*_c2_(*T*) at low temperatures with the experimentally determined *H*_c2_(0) < *H*_c2_^orb^(0) are signatures of the presence of spin-paramagnetic pair-breaking effect. As shown in Fig. 3d and Supplementary Fig. 4, unfortunately, the temperature range of our *μ*_0_*H*_c2_(*T*) data is quite limited so a reliable experimental *μ*_0_*H*_c2_(0) cannot be extracted. Under such a circumstance, we cannot judge the relative importance of the orbital effect versus spin-paramagnetic effect in the pair-breaking process under magnetic fields. Further investigations are required to address this important issue.

### Possible exotic superconducting state under magnetic field

EuTe_2_ under pressure becomes a magnetic superconductor and the interplay between superconductivity and magnetism becomes an interesting issue to pursue. Owing to the large *μ*_0_*H*_c2_ in EuTe_2_, the superconducting state can survive in the spin-flop state with a net FM component. This is quite unusual compared to the conventional BCS superconductors with a spin-singlet pairing state where superconductivity and magnetism are mutually exclusive. In contrary, some exotic magnetic superconductors have been discovered to display unconventional pairing states. For example, in the U-based compounds UGe_2_, UCoGe, UTe_2_ showing the coexistence of itinerant-electron magnetic orders and superconductivity, spin-triplet pairing state has been proposed^9,10,16,18^. On the other hand, the Eu-containing iron-based high-*T*_c_ superconductors have been found to exhibit many peculiar properties associated with the interplay of magnetism and superconductivity including the observations of spontaneous vortex state, the domain Meissner effect, and a vortex-antivortex state in EuFe_2_(As,P)_2_^41^ and Eu(Fe,Rh)_2_As_2_^42^, as well as the superconductivity-driven ferromagnetism and spin manipulation using vortices in EuRbFe_4_As_4_^43^. The discontinuous change of *μ*_0_*H*_c2_(*T*) at the spin-flop transition and the corresponding change of *μ*_0_*H*_c2_(0) around *P*_c_ observed in this study indicate the impact of magnetism on superconductivity. Thus, possible exotic pairing state and/or unconventional superconducting properties deserve further studies in EuTe_2_.

In summary, we report the emergence of superconductivity with *T*_c_ ≈ 3 K at *P*_c_ ≈ 6 GPa and concomitant enhancement of AF transition temperature in the EuTe_2_ single crystal. The superconducting state possess an unusually large upper critical field so that it can survive in the spin-flop state with a net FM component. Our findings establish the pressurized EuTe_2_ as a rare magnetic superconductor that not only demonstrates a mutual promotion between superconductivity and antiferromagnetism but also possesses a possible exotic pairing state.

## Methods

### Single-crystal growth and characterizations at AP

Single-crystal EuTe_2_ used in this work were grown by using the self-flux method^19^. Starting materials Eu (99.9%) and Te (99.999%) shots were mixed in the molar ratio of 1: 10, put into an alumina crucible and then sealed in an evacuated quartz tube. The assembly was slowly heated up to 850 °C in 100 h and held for 3 days in a furnace, then cooled to 450 °C slowly at a rate of 2 °C/h. The assembly was taken out of the furnace and put into high-speed centrifuging immediately at this temperature to remove the excess flux. Crystals with black shiny metallic luster were obtained. The typical size of crystals is ~2.0 × 1.0 × 0.5 mm^3^ and the quality was examined on a Bruker D8 single crystal X-ray diffractometer (SXRD) with Mo K_α_ (*λ* = 0.71073 Å) at room temperature. The diffraction patterns can be well indexed by a tetragonal phase with space group I4/mcm (No. 140). The obtained lattice parameters *a* = 6.9711(3) Å and *c* = 8.1800(5) Å are close to those in literature^19^. The electrical resistivity at AP was measured by standard four-probe method with the current applied along the *c*-axis on a Physical Property Measurement System (PPMS, Quantum Design Inc.).

### High-pressure measurements

Electrical resistivity, ac magnetic susceptibility and ac specific heat were measured under pressures up to 12 GPa by using a palm-type cubic anvil cell (CAC) apparatus in the Synergic Extreme Condition User Facility (SECUF). Electrical resistivity under pressures was measured with the standard four-probe method on two samples labeled as #1 and #2 in two separated runs up to 11.5 and 6.6 GPa. AC magnetic susceptibility *χ*_ac_(*T*) was measured with the mutual reduction method. Sample #3 with dimensions of ~1.0 × 0.25 × 0.1 mm^3^ together with a piece of Pb of size ~1.1 × 0.25 × 0.15 mm^3^ were put in the same detecting coil for the *χ*_ac_(*T*) measurements under pressures up to 11.5 GPa. By comparing the diamagnetic signals of EuTe_2_ and Pb, the superconducting shielding volume fraction of EuTe_2_ can be estimated. High-pressure ac specific heat of sample #4 with dimensions of ~0.53 × 0.44 × 0.09 mm^3^ was measured by the 2*ω* technique. Heater power is modulated by a frequency *ω* and the temperature oscillation of the sample at a frequency 2*ω* is measured by lock-in amplifier (SR830 Stanford Research Systems). Here, 25 µm chromel wires and 25 µm Au/AuFe (0.07%) thermocouple are used as a heater and thermometer, respectively. Glycerol was employed as the pressure transmitting medium (PTM) for resistivity and ac susceptibility measurements while Daphne 7373 as PTM for ac specific-heat measurements. Pressure values were estimated from the calibration curve determined by the shift of the superconducting transition temperature of Pb at low temperatures. Three-axis compression together with the liquid PTM can ensure excellent hydrostatic pressure environments in CAC^22,23^. High-pressure XRD measurements were performed in a diamond pressure cell (DAC) at the BL15U1 station of the Shanghai Synchrotron Radiation Facility (SSRF), which is supported by the Chinese Academy of Sciences. Silicone oil was used as the PTM and the pressure in DAC was monitored by the ruby fluorescence method up to ~12 GPa.

## Supplementary information

Supplementary Information

## Data Availability

The data that support the findings of this study are available from the corresponding authors upon reasonable request.
