# Supplementary material for: Pressured-induced superconducting phase with large upper critical field and concomitant enhancement of antiferromagnetic transition in EuTe2
Source: Nat Commun. 2022 May 27;13:2975. doi: 10.1038/s41467-022-30718-5 (PMC9142537; doi:10.1038/s41467-022-30718-5)
Supplement: Supplementary file 1 — Supplementary Information [file 41467_2022_30718_MOESM1_ESM.pdf]

Supplementary Information

Pressured-induced superconducting phase with large upper critical field and concomitant enhancement of antiferromagnetic transition in $EuTe_2$

P. T. Yang[1,2=], Z. Y. Liu[1=], K. Y. Chen[1,2], X. L. Liu[3], X. Zhang[3], Z. H. Yu[3], H. Zhang[1,2], J. P. Sun[1,2,4], Y. Uwatoko[5], X. L. Dong[1,2,4], K. Jiang[1,2,4], J. P. Hu[1,2], Y. F. Guo[3*], B. S. Wang[1,2,4*], and J.-G. Cheng[1,2*]

[1]*Beijing National Laboratory for Condensed Matter Physics and Institute of Physics, Chinese Academy of Sciences, Beijing 100190, China*

[2]*School of Physical Sciences, University of Chinese Academy of Sciences, Beijing 100190, China*

[3]*School of Physical Science and Technology, Shanghai Tech University, Shanghai 201210, China*

[4]*Songshan Lake Materials Laboratory, Dongguan, Guangdong 523808, China*

[5]*Institute for Solid State Physics, University of Tokyo, Kashiwa, Chiba 277-8581, Japan*

= These authors contributed equally to this work.

Correspondence should be addressed to B.S.W. (bswang@iphy.ac.cn) or Y.F.G. (guoyf@shanghaitech.edu.cn) or J.G.C. (jgcheng@iphy.ac.cn).

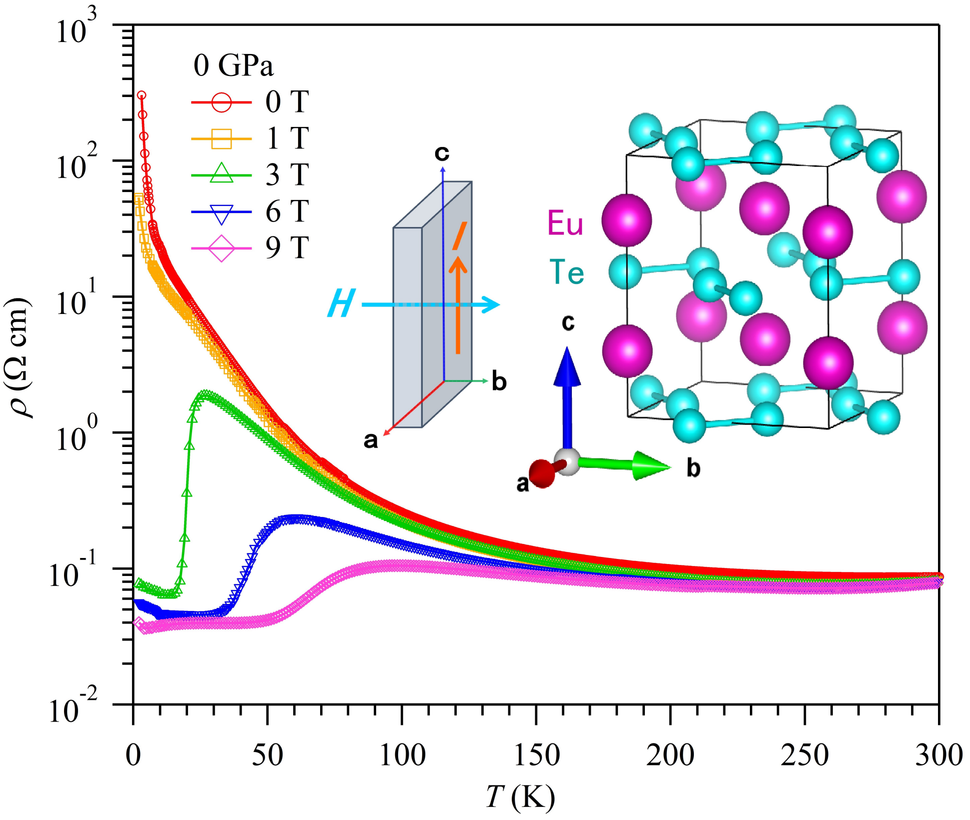

Supplementary Figure 1. Resistivity of $EuTe_2$ at ambient pressure (AP). Temperature dependence of resistivity ρ(T) under various magnetic fields for $EuTe_2$ single crystal at ambient pressure. Inset shows the crystal structure and sample configuration for resistivity measurements. The ρ(T) at 0 T shows a semiconducting behavior in the whole temperature range. When an external magnetic field of 3 T is applied perpendicular to the c-axis, the ρ(T) drops suddenly by more than one order of magnitude upon cooling below $T_m \approx 25$ K, which corresponds to the transition temperature to the low-resistivity metallic state. As a result, a large negative magnetoresistance appears below T_m. With increasing magnetic fields, T_m shifts quickly to higher temperatures and reaches about 90 K under 9 T, consistent with the reported results in Phys. Rev. Mater. 4, 013405 (2020).

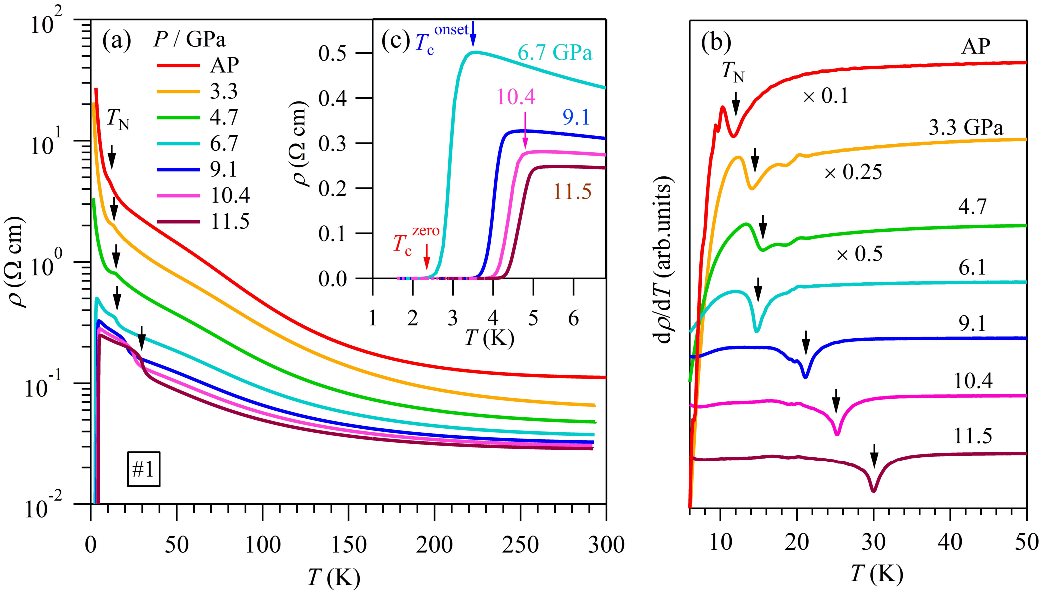

Supplementary Figure 2. High-pressure resistivity of $EuTe_2$ sample #1. (a) $\rho(T)$ and (b) its derivative $d\rho/dT$ under various pressures up to 11.5 GPa for $EuTe_2$ (#1); the arrows indicate the antiferromagnetic (AF) transition. (c) The low-T $\rho(T)$ data at pressures above 6.7 GPa highlighting the evolution of the superconducting transition with the onset and zero-resistance temperatures, T_c^{onset} and T_c^{zero}, marked by the arrows.

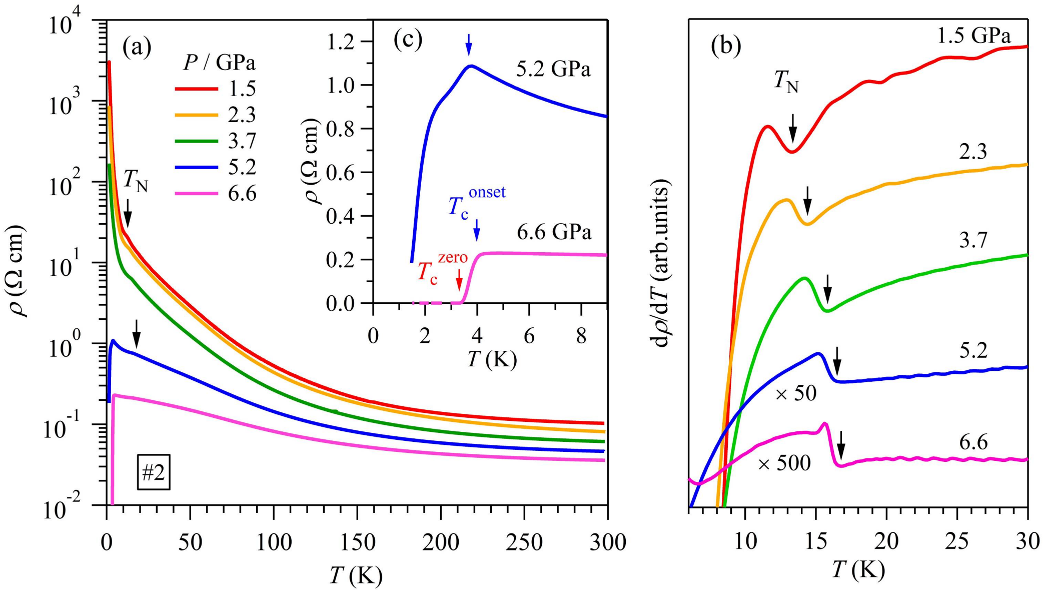

Supplementary Figure 3. High-pressure resistivity of $EuTe_2$ sample #2. (a) $\rho(T)$ and (b) its derivative $d\rho/dT$ under various pressures up to 6.6 GPa for $EuTe_2$ (#2); the arrows indicate the AF transition. (c) The low-T $\rho(T)$ data to show the superconducting phase transition with the T_c^{onset} and T_c^{zero} marked by the arrows.

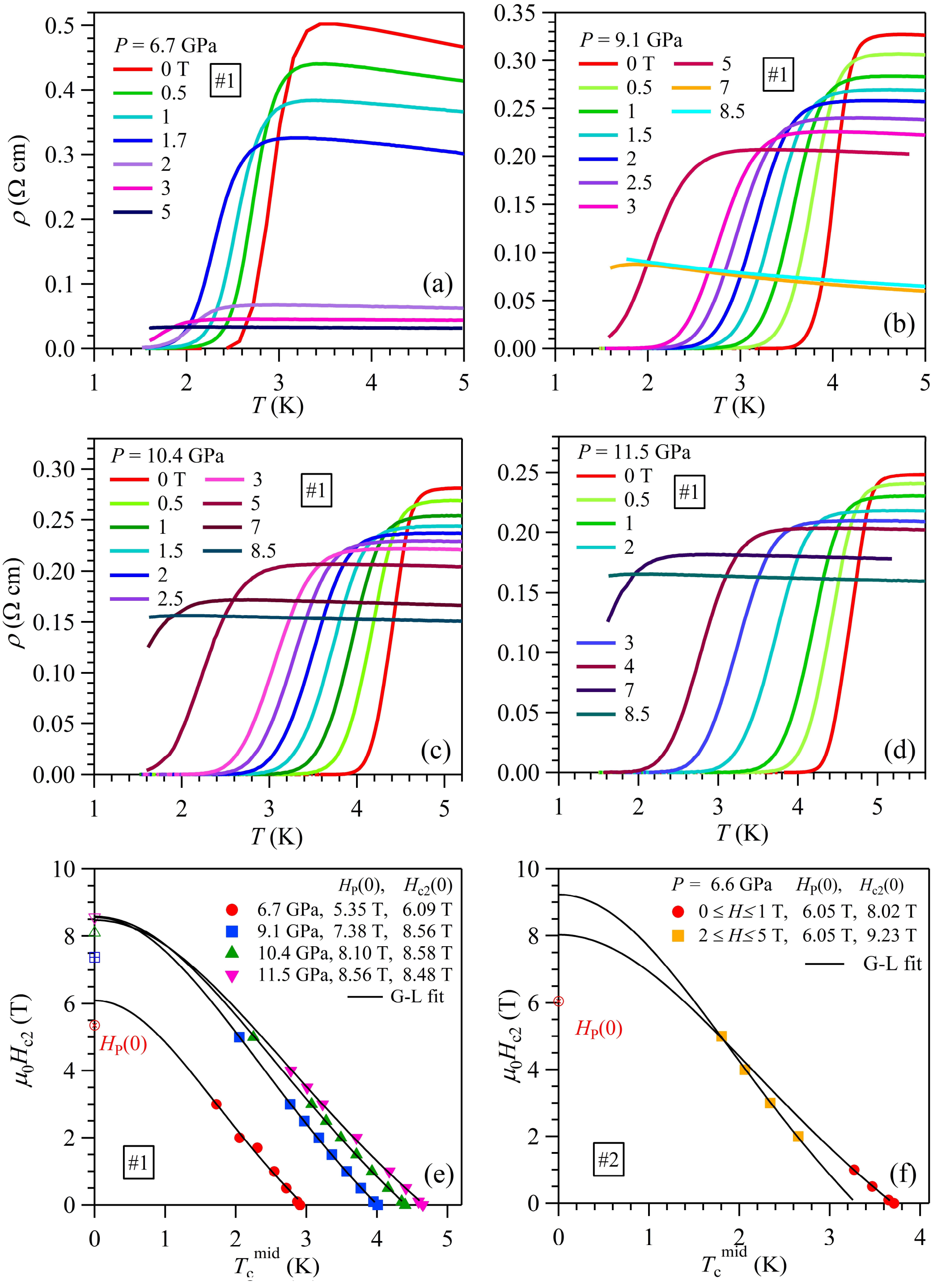

Supplementary Figure 4. Evolution of upper critical field of $EuTe_2$. Low-temperature $\rho(T)$ under different fields for $EuTe_2$ (#1) at various pressures (a) 6.7 GPa, (b) 9.1 GPa, (c) 10.4 GPa, and (d) 11.5 GPa, respectively; Temperature dependence of the upper critical field $\mu_0H_{c2}(T)$ for (e) $EuTe_2$ (#1) and (f) $EuTe_2$ (#2). The empirical Ginzburg-Landau (G-L) equation $\mu_0H_{c2}(T) = \mu_0H_{c2}(0)[1-(T/T_c)^2]/[1+(T/T_c)^2]$ was employed to extract the zero-temperature $\mu_0H_{c2}(0)$ given in (e, f) for comparison.

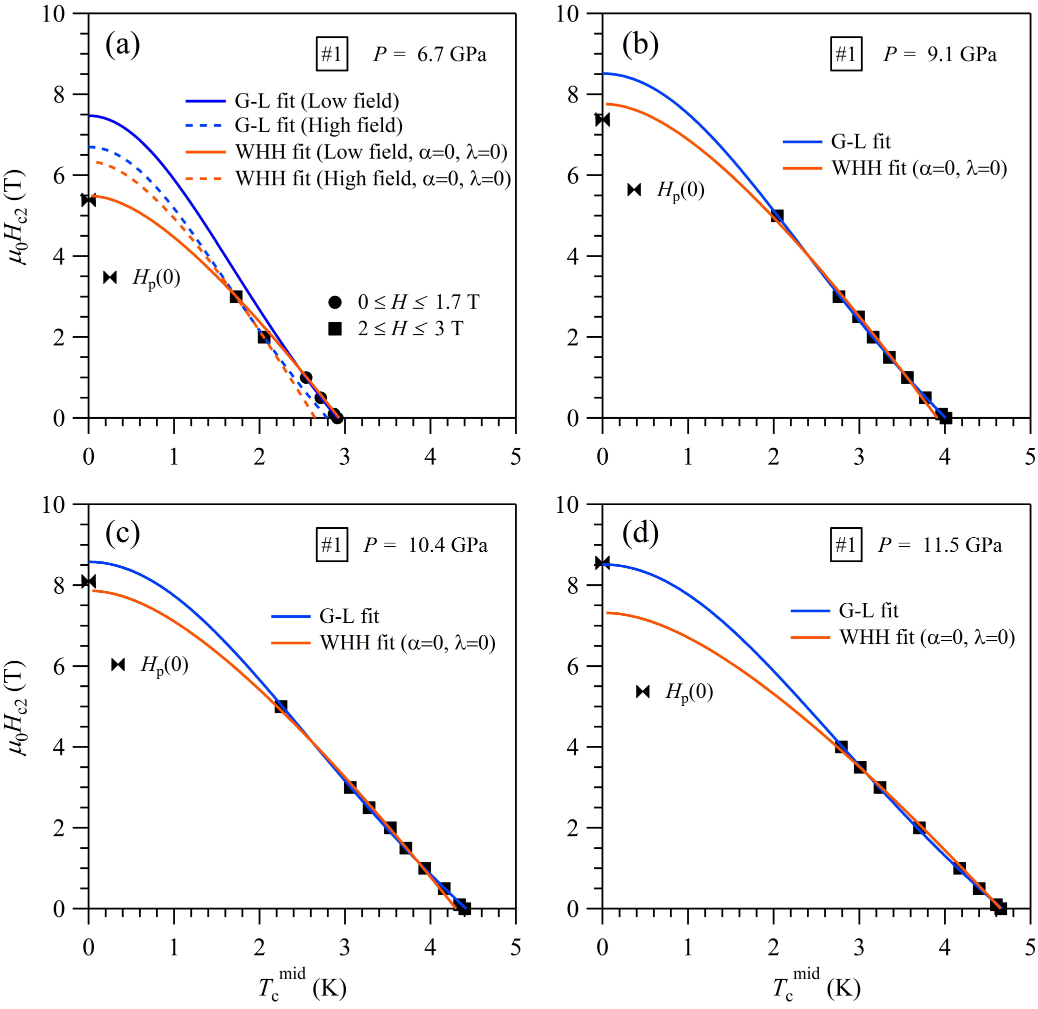

Supplementary Figure 5. **Comparison between the G-L and WHH fittings.** Temperature dependence of $\mu_0 H_{c2}(T)$ for sample #1 at various pressures (a) 6.70 GPa; (b) 9.1 GPa; (c) 10.4 GPa; (d) 11.5 GPa. The blue and red solid lines represent the fitting to the Ginzburg-Landau (G-L) equation and Werthamer-Helfand-Hohenberg (WHH) model, respectively. The broken lines represent the G-L equation and WHH model fitting to the data points in the high-field regime for sample #1 at 6.7 GPa.

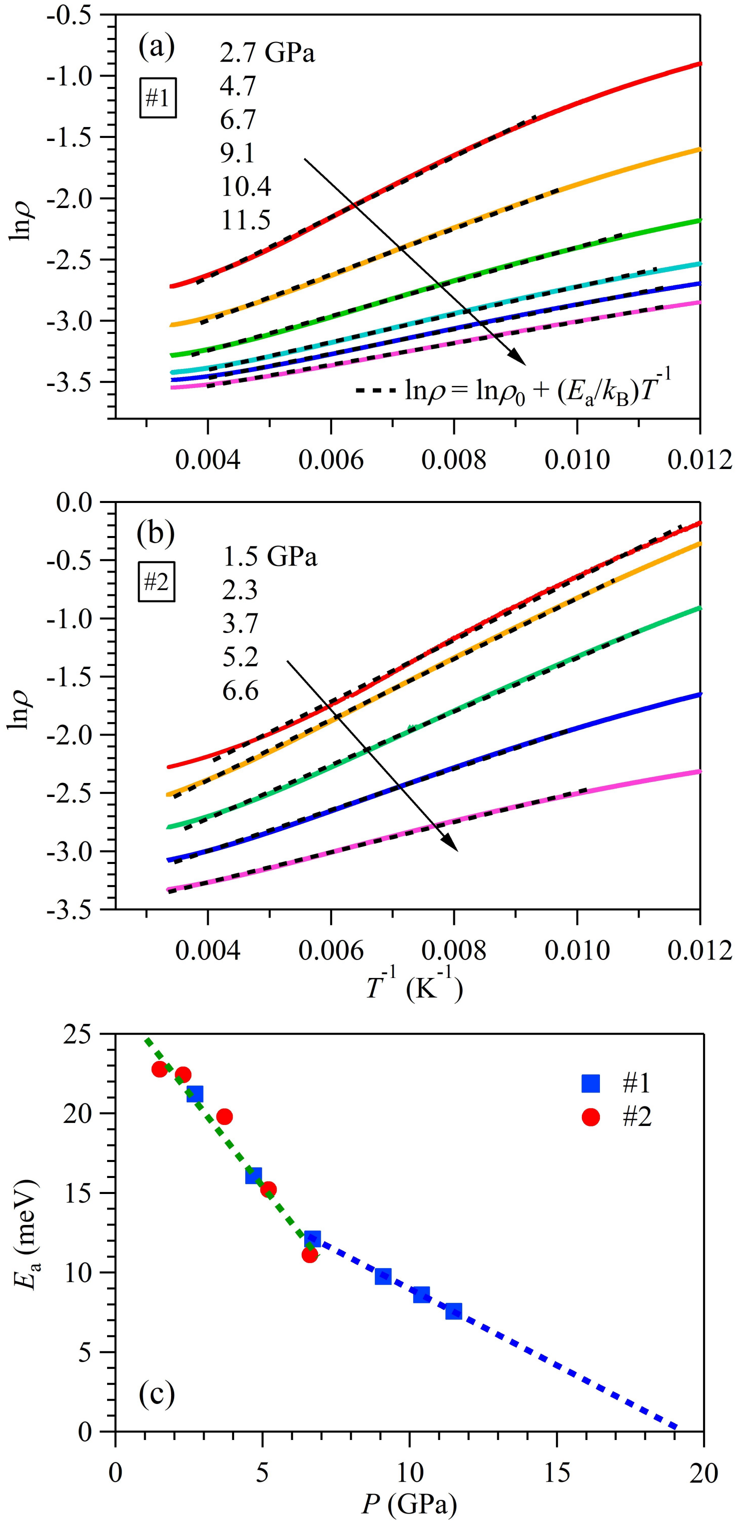

Supplementary Figure 6. **Evolution of the activation gap for $EuTe_2$ under high pressure.** The analysis of high-temperature resistivity data (80-260 K) for (a) #1 and (b) #2 by using the thermal activation model $\rho(T) = \rho_0\exp(E_a/k_BT)$, where ρ_0 is a constant and k_B is Boltzmann constant; (c) the activation energy E_a quickly decreases from ~ 23 meV at AP to ~12.2 meV at P_c, and then slowly to ~7 meV at 11.5 GPa. The metallization of $EuTe_2$ accompanying the semiconducting-gap closure may appear at about 20 GPa according to the linear extrapolation of $E_a(P)$ in (c).

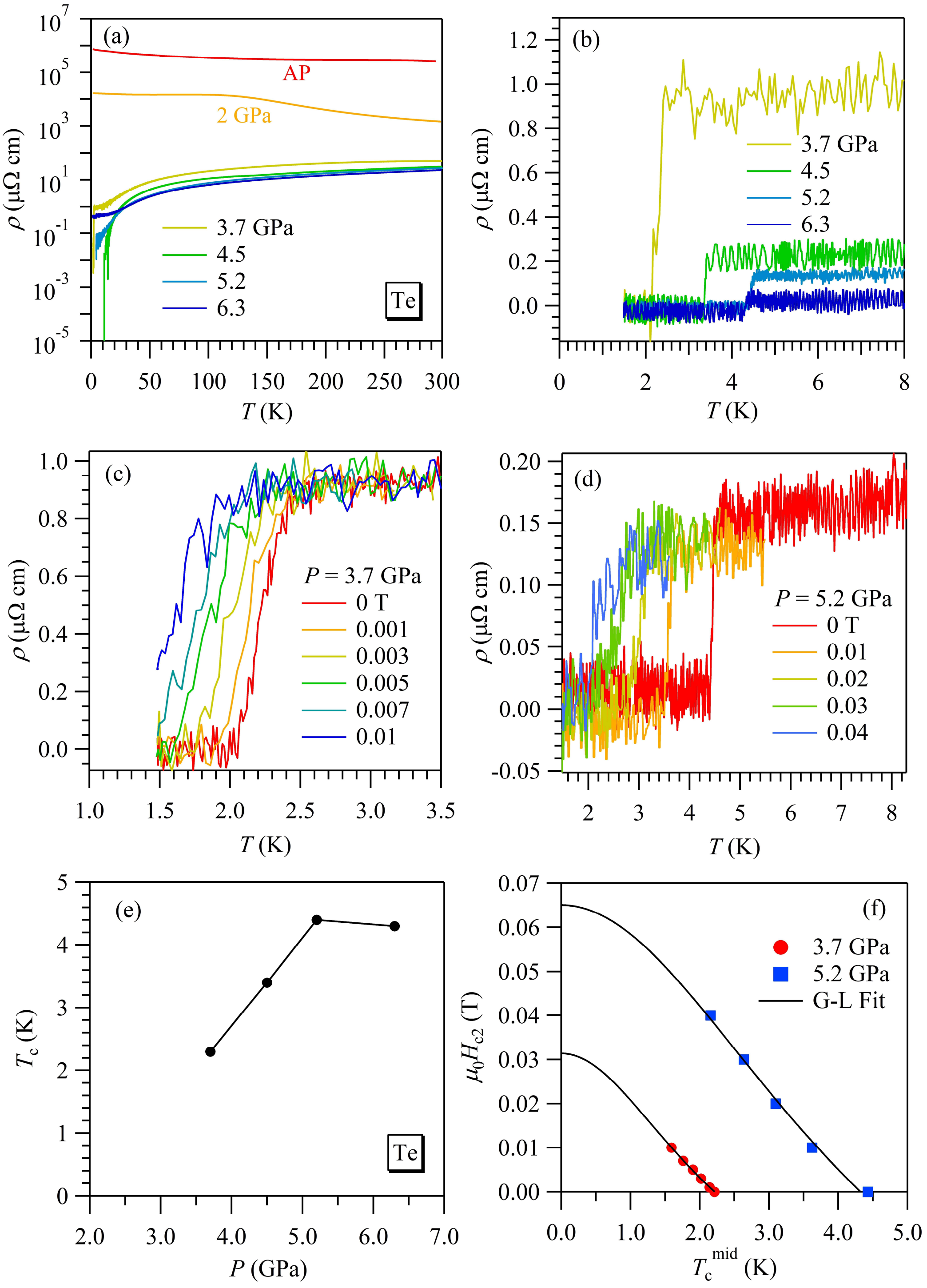

Supplementary Figure 7. High-pressure transport properties of Te. (a) Temperature dependence of $\rho(T)$ under various pressures up to 6.3 GPa for single-crystal Te; (b) Low-temperature $\rho(T)$ of Te under various pressures of 3.7, 4.5, 5.2 and 6.3 GPa; (c, d) Field-dependent $\rho(T)$ under various pressures at 3.7 and 5.2 GPa; (e) Pressure dependence of T_c for Te; (f) Temperature dependence of the upper critical field $\mu_0 H_{c2}(T)$ fitted with the empirical Ginzburg-Landau (G-L) equation at 3.7 and 5.2 GPa.
